# Closing Editorial: From intercultural competence to intercultural sensitivity in medical education

**DOI:** 10.15694/mep.2018.0000068.1

**Published:** 2018-03-23

**Authors:** Janusz Janczukowicz, Petra Verdonk

**Affiliations:** 1 Medical University of Lodz; 2 Vrije Universiteit- Amsterdam

**Keywords:** diveristy, intercultural competence, intercultural sensitivity

## Abstract

This article was migrated. The article was marked as recommended.

This editorial summarises the MedEdPublish Special Issue on Diversity and encourages further research and educational cooperation in this area.

## Introduction

In the opening editorial of this MedEdPublish special issue we have suggested a framework adding structure to the complex discourses on diversity in medical education based on three distinct levels: fixing the numbers referring to the structure of the students’ and faculty’s cohorts in education, fixing the institutions, understood as focusing on educational climate and organizational culture, and fixing the knowledge with focus on generating new knowledge, and providing the diversity perspective to health professions medical curricula.(1)

This is really important to see that in response to call for papers to our special issue we have received manuscripts covering all the areas of the suggested framework written by the authors representing diverse health and educational professions from diverse countries. The topics presented include students’ evaluation of an undergraduate diversity training, ethnic diversity of UK dentistry, cultural context of veterinary education, methodology of gender medicine teaching, improving access and equity in higher education, training for ethical and comprehensive care of patients living with HIV/AIDS, gender differences in feedback giving and receiving strategies, gender based violence, factors limiting inclusion of students with disabilities, and measures taken to ensure that disabled learners do not face unnecessary barriers to their careers, design and implementation of LGBT health curriculum, dimensions of perceptions of tattooed health professional, differences in women’s health and maternity care training, and integration of diversity aspects into health professions study programmes. Obviously, all three levels of our framework presented in the published papers have very strong and complex relationships and to fully understand them, including the hidden issues of power and privilege, the further analysis should be performed from the intersectional perspective.

## Conclusions

A major issue worth addressing in this reflection is related to the significant lack of evaluation of diversity-related education interventions at levels higher than participants’ satisfaction. While multiple published resources describe newly implemented curricula on diversity, usually they report evaluation based on students’ surveys or focus group. Not only such evaluation doesn’t necessarily translate in to particular educational outcomes but at the same time there is no agreement on the reliable assessment methods of diversity-related knowledge, skills and attitudes. Furthermore, evaluation of effectiveness of curricula at levels of individual patients and societies is even sparser. We need to keep in mind that especially in the scope of diversity-equality-equity even the carefully designed interventions may be not only not effective but also counter effective or generating stress as in the classic Jane Elliot primary education experiment “Blue- Eyes- Brown Eyes”. (2) The main aim of this paragraph is to call for focusing research in this area on higher levels evaluation of diversity-related curricula, optimally- applying the action research cyclical and participatory approach.

Teaching and learning will never be effective if students consciously or subliminally identify tensions between the formal curricular content and the hidden curriculum. To at least partially address the issue of tensions between the explicit diversity-related curricular elements implemented by diversity champions and the hidden curriculum embedded in the institutional culture, all preclinical clinical and preclinical faculty should be involved in longitudinal educational strategies implicitly implemented at all levels of under- and postgraduate education. With the significant shift of Evidence Based Medicine from the purely quantitative, statics-based strategy of recommendations to the individualised approach grounded in patient’s socio-cultural context and shared decisions making respecting patient’s values, emerges a clear need not only for the diverse population of health professions teachers but also the diverse population of assessors able to properly address all dimensions and intersections of diversity.(3,4) Furthermore, we need to remember that providing students with teaching on certain domains (in this case- diversity), without assessing this domain while assessing the other competencies conveys a clear subliminal message to the learner: “this is not really important, otherwise they would asses it”.

Moreover, in the current era of globalisation and increased students’ mobility multiple universities are facing challenges of providing education to extremely multicultural cohorts of student’s for the first time migrating outside their countries and starting their difficult medical studies with no time for understanding the new cultural context, including the significantly new educational and professional expectations of their news schools. Such students struggling with the cultural dissonance between their own values and the new environment often dissociate academic and medical professional values and require effective support and mentoring. (5)

Finally, the pure “numbers” can be very misleading if not harmful for the underrepresented groups of students. While multiple resources indicate widespread admission inequalities and the divisive in ethnicity and social class elitism of certain universities, fixing the admission numbers without providing the appropriate educational and career monitoring and support may lead to serious negative consequences and increasing the alienation of minority students and students from disadvantaged backgrounds expected to follow the dominant educational culture and climate of the institution. As a result, the optimistic indicators of inclusive access may not translate into the consecutive expected social mobility of minority students and graduates. While the currently used social mobility indices are very often ambiguous, application of in-depth analytical tools can reveal the painful truth about many institutions proud of their superficial diversity. (6)

The title of this editorial emphasises the need for transformation from providing students with factual knowledge about all dimensions of diversity -obviously necessary but at the same time often enhancing stereotypes and increasing the process of “othering” (e.g. 7), towards developing intercultural skills based on empathy, sensitivity and self-reflection, including the capability to identify own implicit stereotypes. To give an example of complexities and tensions between teaching factual knowledge, developing students’ sensitivity and providing safe educational environment, we may present the case of medical standards of providing care to Jehovah’s Witnesses patients. Majority of countries have well designed regulations regarding the appropriate procedures in case of patients refusing blood transfusions depending on their consciousness, age and the presence or absence of the advance directive. While teaching this factual knowledge we need to be aware that both the students’ and teachers’ cohorts may include Jehovah’s Witnesses and the way we deliver our teaching instead of providing them with safe environment to present their perspective may increase their segregation and alienation, especially keeping in mind that the aforementioned regulations implemented with the intent to protect patient’s informed consent and safety may be perceived by them as offensive and disrespectful.

While we are definitely at the beginning of the complex road towards fixing the numbers, institutions and knowledge and while we need to challenge our own stereotypes and step out of our own comfort zone like it happens while reading Gloria Wekker’s publications on white innocence and racism grounded deep in societies proud of their multiculturalism (8), we are extremely happy with so many valuable contributions to this special issue of MedEdPublish and hope to meet both the current and the new authors during the forthcoming AMEE conference in Basel. One of the crucial issues we plan to discuss in Basel is the situation of the refugee health professions students and the possibilities of offering them support by individual schools and educational systems.

We hope to meet you in Basel!

## Notes On Contributors

Dr. Janusz Janczukowicz MD, MMEd, PhD (janusz.janczukowicz@umed.lodz.pl) is a head of Centre for Medical Education at the Medical University of Lodz. He is also the Member of the AMEE Executive Committee and the Board Member of the European Institute of Women’s’ Health, with main expertise in professionalism, diversity, social competence and general medical education.

Dr. Petra Verdonk (p.verdonk@vumc.nl) is an occupational health psychologist with a PhD in integrating gender perspective in medical curricula. She is an assistant professor at the department of Medical Humanities at VUMC Amsterdam. Her main areas of expertise and research interest include gender and intersectionality/diversity issues in medical education and public health.

## Declarations

Dr. Janusz Janczukowicz and Dr. Petra Verdonk are guest Theme Editors for AMEE MedEdPublish for the theme of Diversity in Medical Education.
