# Supplementary material for: Closing Editorial: From intercultural competence to intercultural sensitivity in medical education
Source: MedEdPublish (2016). 2018 Mar 23;7:68. [Version 1] doi: 10.15694/mep.2018.0000068.1 (PMC10711942; doi:10.15694/mep.2018.0000068.1)
Supplement: Peer review history

# Reviewer report: Reviewer response for version 1

**DOI:** 10.21956/mep.19535.r28924

**Published:** 2018-04-13

**Authors:** Barbara Jennings

**Affiliations:** 1 Norwich Medical School

This review has been migrated. The reviewer awarded 4 stars out of 5 I really enjoyed reading this article and the papers that made up the special issue on diversity. The authors have reminded us of some important principles:1. Evidence based practice requires data from research studies that go beyond participant / learner satisfaction.2. If we don't assess new areas of a curriculum robustly it won't be perceived as important by our student body3. Power structures need to be considered and we need to ensure that our measurements of diversity are not superficial. Data from undergraduate admissions are one thing but what about subsequent career progression and health outcomes.I will be sharing links from this Themed edition with colleagues - it might have been helpful to have the contributing papers summarised and cited in this closing editorial?

Reviewer Expertise:

NA

No decision status is available

# Reviewer report: Reviewer response for version 1

**DOI:** 10.21956/mep.19535.r28927

**Published:** 2018-03-24

**Authors:** Gary D. Rogers

**Affiliations:** 1 Griffith University

This review has been migrated. The reviewer awarded 5 stars out of 5 Congratulations Janusz and Petra on a very successful themed edition of MedEdPublish. Many of the authors, along with Subha's review, have pointed to the importance of effective preparation and continuing development of educators in order to ensure fulfill the 'fixing the institutions' part of the framework. As guest editor of the next themed edition, which will focus more broadly on the development of health professional educators, I would welcome submissions reporting practical attempts to educate and up-skill educators in relation to diversity or theoretical formulations that might underpin such development.

Reviewer Expertise:

NA

No decision status is available

# Reviewer report: Reviewer response for version 1

**DOI:** 10.21956/mep.19535.r28925

**Published:** 2018-03-23

**Authors:** Trevor Gibbs

**Affiliations:** 1 AMEE

This review has been migrated. The reviewer awarded 5 stars out of 5 A very important theme that attracted some very important papers, many of which explored the different elements of diversity. The papers however do point out the difficulties in the issue of diversity and that we cannot be complacent in thinking that we are effectively dealing with it- we still have a lot of work to do. Well done in running this theme

Reviewer Expertise:

NA

No decision status is available

# Reviewer report: Reviewer response for version 1

**DOI:** 10.21956/mep.19535.r28926

**Published:** 2018-03-23

**Authors:** Subha Ramani

**Affiliations:** 1 Harvard Medical School, Brigham and Women's Hospital

This review has been migrated. The reviewer awarded 5 stars out of 5 The theme editors have proposed a great model for addressing diversity in medical education and this closing editorial is quite thought provoking, I completely agree that numbers is not 'the fix' to this dilemma, attitudes, institutional and professional culture, learning environment are important factors that would impact this area in health professions education. The editors also point out the importance of congruence between the formal and hidden curriculum. A lot of staff development and leadership development would be critical to achieve the outcomes that the editors have highlighted.The world of health professions education- learners, teachers and leaders, need to think about this topic.

Reviewer Expertise:

NA

No decision status is available
